# Genes in S and T Subgenomes Are Responsible for Hybrid Lethality in Interspecific Hybrids between *Nicotiana tabacum* and *Nicotiana occidentalis*

**DOI:** 10.1371/journal.pone.0036204

**Published:** 2012-04-26

**Authors:** Takahiro Tezuka, Wataru Marubashi

**Affiliations:** 1 Graduate School of Life and Environmental Sciences, Osaka Prefecture University, Sakai, Osaka, Japan; 2 School of Agriculture, Meiji University, Kawasaki, Kanagawa, Japan; University of Warwick, United Kingdom

## Abstract

**Background:**

Many species of *Nicotiana* section *Suaveolentes* produce inviable F_1_ hybrids after crossing with *Nicotiana tabacum* (genome constitution SSTT), a phenomenon that is often called hybrid lethality. Through crosses with monosomic lines of *N. tabacum* lacking a Q chromosome, we previously determined that hybrid lethality is caused by interaction between gene(s) on the Q chromosome belonging to the S subgenome of *N. tabacum* and gene(s) in *Suaveolentes* species. Here, we examined if hybrid seedlings from the cross *N. occidentalis* (section *Suaveolentes*)×*N. tabacum* are inviable despite a lack of the Q chromosome.

**Methodology/Principal Findings:**

Hybrid lethality in the cross of *N. occidentalis*×*N. tabacum* was characterized by shoots with fading color. This symptom differed from what has been previously observed in lethal crosses between many species in section *Suaveolentes* and *N. tabacum*. In crosses of monosomic *N. tabacum* plants lacking the Q chromosome with *N. occidentalis*, hybrid lethality was observed in hybrid seedlings either lacking or possessing the Q chromosome. *N. occidentalis* was then crossed with two progenitors of *N. tabacum*, *N. sylvestris* (SS) and *N. tomentosiformis* (TT), to reveal which subgenome of *N. tabacum* contains gene(s) responsible for hybrid lethality. Hybrid seedlings from the crosses *N. occidentalis*×*N. tomentosiformis* and *N. occidentalis*×*N. sylvestris* were inviable.

**Conclusions/Significance:**

Although the specific symptoms of hybrid lethality in the cross *N. occidentalis*×*N. tabacum* were similar to those appearing in hybrids from the cross *N. occidentalis*×*N. tomentosiformis*, genes in both the S and T subgenomes of *N. tabacum* appear responsible for hybrid lethality in crosses with *N. occidentalis*.

## Introduction

Reproductive isolation involves various types of prezygotic and postzygotic barriers in plants [1]. Although these barriers contribute to the formation of species, they are obstacles for plant breeders, especially in breeding programs involving wide hybridization. Hybrid plants from normal parents often show weak growth or death before maturity. Several terms are used to describe this phenomenon, including hybrid lethality, hybrid weakness, hybrid necrosis, and hybrid inviability. Hybrid lethality is a type of postzygotic barrier and is observed in certain cross combinations in numerous plant species, including *Nicotiana* sp. [2], *Oryza* sp. [3], [4], *Phaseolus vulgaris* [5], *Lactuca* sp. [6], and *Arabidopsis thaliana* [7].

The genus *Nicotiana* comprises 76 species that are predominantly distributed in the Americas and Australia [8]. This complex genus has attracted the attention of many researchers investigating evolutionary processes. The most well-studied and characterized species in this genus is cultivated tobacco, *Nicotiana tabacum* (2n = 48, SSTT), which belongs to section *Nicotiana*. *N. tabacum* is a natural allotetraploid (amphidiploid) that originated by interspecific hybridization of *N. sylvestris* (2n = 24, SS) with *N. tomentosiformis* (2n = 24, TT) and subsequent chromosome doubling [9]–[16]. Each chromosome of *N. tabacum* is lettered alphabetically (A–Z, excluding X and Y); chromosomes A–L and M–Z belong to the T and S subgenomes, respectively. A complete set of 24 monosomic lines of *N. tabacum* (Haplo-A–Z), which lack a certain chromosome, has been established in the genetic background of ‘Red Russian’ and is useful for locating genes on specific chromosomes [17]–[19]. Monosomic analyses have identified one *N. tabacum* chromosome containing a gene or genes that trigger hybrid lethality [20]–[22].

Hybrid lethality in the genus *Nicotiana* is currently classified into four types based on surface symptoms, as follows: Type I, browning of shoot apex and root tips; Type II, browning of hypocotyl and roots; Type III, yellowing of true leaves; and Type IV, formation of multiple shoots [2]. Although several methods to overcome hybrid lethality have been reported in the genus *Nicotiana*, their effectiveness is dependent on the type of hybrid lethality encountered. For example, hybrid lethality of Types I, II, and III is temperature sensitive; i.e., hybrid lethality is observed at 28°C, but not at elevated temperatures of approximately 36°C. In contrast, Type IV lethality is not suppressed at elevated temperatures [2].

Our previous studies indicate that many species in *Nicotiana* section *Suaveolentes* produce inviable hybrids after crosses with *N. tabacum* [20]–[22]. Section *Suaveolentes* includes 26 species, most of which are endemic to Australia, although three species are found in other locations. For example, *N. debneyi* is distributed in Eastern Australia, New Caledonia, and Lord Howe Island, while *N. fragrans* is distributed in New Caledonia, and the Loyalty, Tonga, and Marquesas Islands [23], [24]. One species, *N. africana*, is distributed in Namibia (South-West Africa), and represents the only *Nicotiana* species discovered in Africa to date [25]. Species in section *Suaveolentes* are geographically isolated from the majority of species in other sections, which are distributed in the Americas.

All species in section *Suaveolentes* are allotetraploids and comprise an almost complete aneuploid series of n = 16–24, with only n = 17 yet to be identified. Section *Suaveolentes* is considered to have originated from a single polyploid event approximately 10 mya, followed by speciation to produce the species known today [26]. This hypothesis is supported by recent studies indicating that section *Suaveolentes* is a monophyletic group based on analyses of internal transcribed spacer (ITS) regions [14], plastid genes [27], and nuclear-encoded chloroplast-expressed glutamine synthetase (ncpGS) [16]. Furthermore, progenitors of this section have been identified based on sequence analyses of ncpGS; the maternal progenitor is *N. sylvestris* and the paternal progenitor is section *Trigonophyllae* [16]. One group of *Suaveolentes* species is genetically distant from *N. tabacum*, even though they share the same maternal progenitor, *N. sylvestris* [14], [16], [27]. Although *Suaveolentes* species are valuable as sources of disease resistance [28]–[30] and cytoplasmic male sterility [31], [32], the occurrence of hybrid lethality represents a barrier for introducing desirable characteristics into *N. tabacum* by interspecific crosses.

After crosses with *N. tabacum*, nine species in section *Suaveolentes*, namely *N. africana*, *N. debneyi*, *N. excelsior*, *N. goodspeedii*, *N. gossei*, *N. maritima*, *N. megalosiphon*, *N. suaveolens*, and *N. velutina*, produce inviable hybrids, whereas only *N. benthamiana* and *N. fragrans* generate 100% viable hybrids [20]–[22], [33]. Hybrid lethality observed in all crosses at 28°C is Type II and is suppressed at temperatures of 34–36°C. We also previously conducted genetic analyses of hybrid lethality, which revealed that the Q chromosome in the S subgenome of *N. tabacum* encodes one or more genes leading to hybrid lethality in crosses using the nine species of section *Suaveolentes* [20]–[22]. Based on the existence of a single origin of section *Suaveolentes*, we proposed that many species in section *Suaveolentes* share the same gene(s) triggering hybrid lethality by interaction with gene(s) on the Q chromosome [22].

In the present study, we examined hybrid lethality in crosses between *N. occidentalis*, which belongs to section *Suaveolentes*, and *N. tabacum*. *N. occidentalis* is closely related to other Australian species in section *Suaveolentes* and is distant from *N. tabacum* based on recently constructed phylogenetic trees [14], [16], [27]. Although Ternovskii et al. [34] reported that hybrid seedlings from the cross *N. occidentalis* ♀×*N. tabacum* ♂ die without forming roots at the cotyledonary stage, the nature of the lethality was incompletely described. Here, based on phenotypic and genetic analyses, we demonstrate that *N. occidentalis* exhibits a distinct type of hybrid lethality after crosses with *N. tabacum* than that observed with the nine *Suaveolentes* species examined in our previous studies.

## Results

### Temperature-sensitive lethality in hybrid seedlings of *N. occidentalis* ♀×*N. tabacum* ♂

Reciprocal crosses between *N. occidentalis* and *N. tabacum* (cultivars ‘Red Russian’ and ‘Samsun NN’) were carried out using conventional cross-pollination (Table 1). When *N. occidentalis* was used as the paternal parent, flowers of *N. tabacum* dropped approximately 7 days after pollination. The ovaries and ovules of these flowers did not enlarge, suggesting that fertilization did not occur. In contrast, when *N. occidentalis* was used as the maternal parent, seeds that germinated well at 28°C under *in-vitro* conditions were obtained.

**Table 1:** Conventional crossings between *N. occidentalis* and *N. tabacum*.

Cross combination	No. of flowers pollinated	No. of capsules obtained	No. of seeds sown	No. of hybrids obtained
*N. occidentalis* ♀×‘Red Russian’ ♂	20	19	340	305
‘Red Russian’ ♀×*N. occidentalis* ♂	21	0	–	–
*N. occidentalis* ♀×‘Samsun NN’ ♂	20	12	259	215
‘Samsun NN’ ♀×*N. occidentalis* ♂	20	0	–	–
*N. occidentalis*	–	–	140	136
‘Red Russian’	–	–	140	137
‘Samsun NN’	–	–	140	129

**Figure 1:**
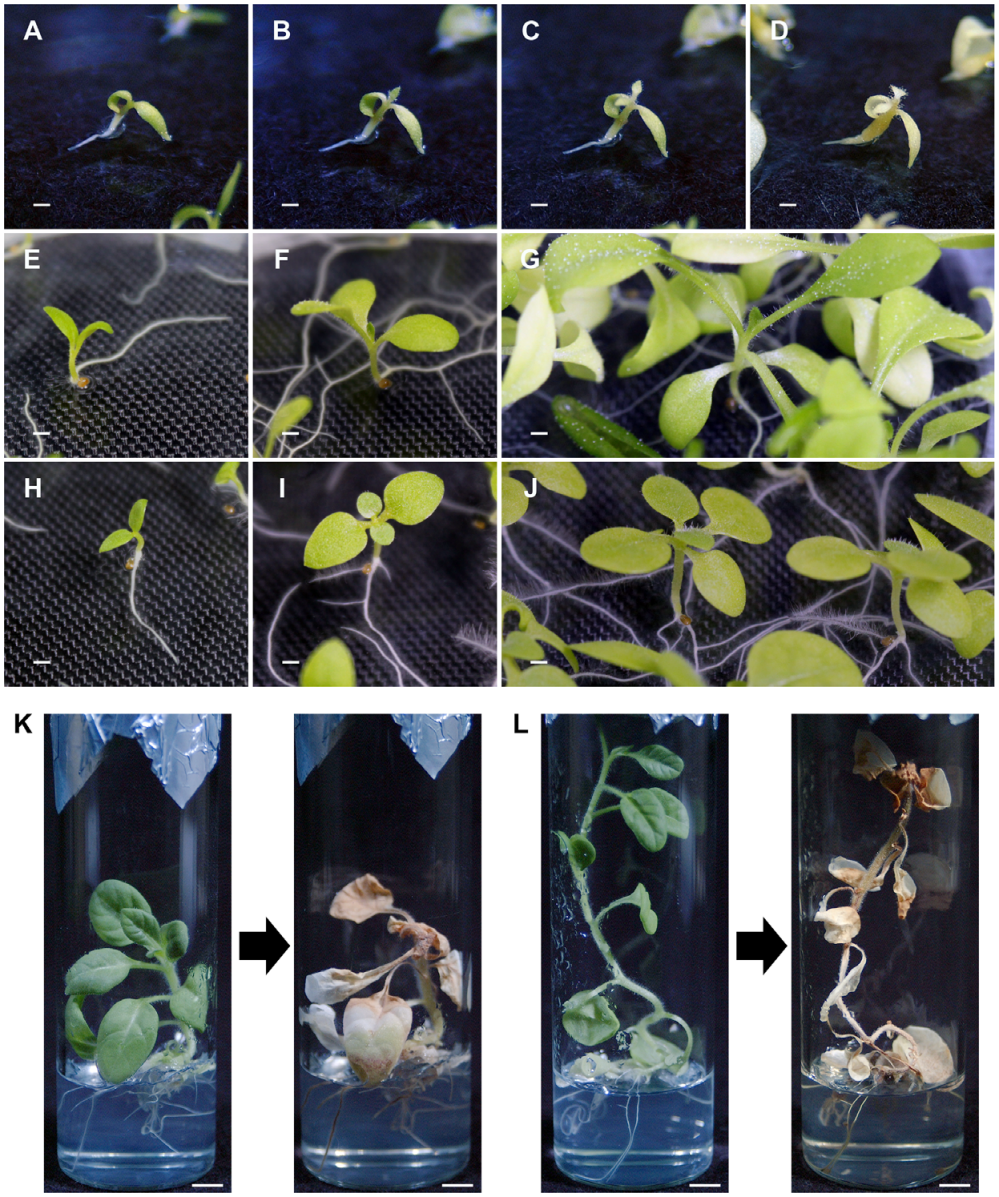
Lethal symptoms observed in hybrid seedlings from crosses between *N. occidentalis* and *N. tabacum*. (A–D) Hybrid seedlings from the cross *N. occidentalis* ♀×*N. tabacum* ‘Red Russian’ ♂ at 24°C. Although hybrid seedlings at 3 days after germination (DAG) exhibited apparently normal growth, root growth nearly stopped (A). The color of cotyledons and hypocotyl faded beginning from 8 DAG (B), continued to advance at 10 DAG (C), with the seedlings turning completely brown and dying by 30 DAG (D). (E–G) *N. occidentalis* grown at 24°C. *N. occidentalis* grew normally and did not show lethal symptoms at 3 (E), 10 (F), or 30 DAG (G). (H–J) ‘Red Russian’ grown at 24°C. ‘Red Russian’ also grew normally and did not display lethal symptoms at 3 (H), 10 (I), or 30 DAG (J). (K, L) Hybrid seedlings from the cross (*N. tabacum* Haplo-Q×*N. tabacum* ‘Samsun NN’) ♀×*N. occidentalis* ♂. Hybrid seedlings possessing (K) and lacking (L) the Q chromosome died after being transferred from 34°C (left panels) to 24°C (right panels). Scale bars = 1 mm (A–J) and 5 mm (K, L).

Hybrid seedlings from the cross *N. occidentalis* ♀×*N. tabacum* ♂ were grown *in vitro* at 24, 28, and 34°C (Table 2). At 24°C, hybrid seedlings grew normally and developed cotyledons and roots by approximately 3 days after germination (DAG), although root growth stopped at this stage (Figure 1A). The color of cotyledons and the hypocotyl began to fade approximately 8 DAG (Figure 1B, C), with hybrid seedlings dying at about 30 DAG (Figure 1D). A characteristic symptom of hybrid lethality was the fading of shoot color. As this symptom was distinct from those of the four known types of *Nicotiana* hybrid lethality [2], the type of lethality observed here was designated as Type V.

**Table 2:** Viability of hybrid seedlings generated from crosses between *N. occidentalis* and *N. tabacum* at different temperatures.

		No. of hybrids			
Cross combination	Temperature (°C)	Total	Viable	Inviable	Lethality type^a^
*N. occidentalis* ♀×‘Red Russian’ ♂	24	80	0	80	V
	28	205	0	205	V
	34	20	20	0	–
*N. occidentalis* ♀×‘Samsun NN’ ♂	24	74	0	74	V
	28	121	0	121	V
	34	20	20	0	–
*N. occidentalis*	24	57	57	0	–
	28	59	59	0	–
	34	20	20	0	–
‘Red Russian’	24	57	57	0	–
	28	60	60	0	–
	34	20	20	0	–
‘Samsun NN’	24	56	56	0	–
	28	53	53	0	–
	34	20	20	0	–

a Type V, fading of shoot color.

Hybrid seedlings also displayed Type V lethality at 28°C, although the lethal symptoms appeared later (approximately 30 DAG) than for seedlings grown at 24°C, with the seedlings surviving for a few months (Table 2). In contrast, hybrid seedlings grown at 34°C did not show lethal symptoms for at least 60 DAG (Table 2). When these hybrid seedlings were transferred to 28°C, they ceased growing and died 40–70 days after the transfer. The three parental species, *N. occidentalis*, ‘Red Russian’, and ‘Samsun NN’, grew normally without the appearance of lethal symptoms for at least 60 DAG at 24, 28, and 34°C (Table 2, Figure 1E–J).

### Hybrid seedlings die even when they lack the Q chromosome

*N. tabacum* monosomic plants lacking the Q chromosome, namely F_1_ progeny derived from the cross *N. tabacum* Haplo-Q ♀×‘Samsun NN’ ♂ [35], were crossed with *N. occidentalis* to determine whether the Q chromosome is responsible for hybrid lethality. Monosomic plants were used as maternal parents, as the transmission of the monosomic condition through pollen seldom occurs in *N. tabacum* [36]. When *N. tabacum* was pollinated with *N. occidentalis*, fertilization did not occur, as described above. Therefore, test-tube pollination and ovule culture were performed to overcome this crossing barrier.

Twenty-nine placentas of monosomic plants were pollinated *in vitro*, resulting in 445 enlarged ovules that were cultured at 28°C. The 30 hybrid seedlings obtained from the cultured ovules were further grown at 34°C and then assessed for the presence or absence of the Q chromosome using three Q-chromosome-specific sequence-tagged-site (STS) markers: QCS2, QCS3, and QCS4 [20], [35]. Five of the hybrid seedlings exhibited abnormal morphology and lacked cotyledons and leaves. Because genomic DNA could not be isolated from these five seedlings, they were excluded from further analyses.

Using the Q-chromosome-specific STS markers, the remaining 25 hybrid seedlings were divided into two types: 11 hybrid seedlings possessing the Q chromosome and 14 hybrid seedlings lacking the Q chromosome (Table 3). Both types of hybrid seedlings were cultured for 30 DAG and then transferred from 34 to 28°C. After 40–70 days, both types of seedlings showed lethal symptoms and died (Figure 1K, L). Although a characteristic symptom of hybrid lethality is the fading of shoot color, as reported above, no difference in this symptom was observed between the two types of hybrid seedlings. These results indicated that lack of the Q chromosome does not suppress hybrid lethality in *N. tabacum* ♀×*N. occidentalis* ♂ progeny.

**Table 3:** Relationship between the Q chromosome and hybrid lethality in crosses between *N. tabacum* and *N. occidentalis*.

		No. of hybrids		
Cross combination	STS markers^a^	Total	Viable	Inviable
(Haplo-Q×‘Samsun NN’) ♀×*N. occidentalis* ♂	+	11	0	11
	−	14	0	14

a ‘+’ indicates that Q-chromosome-specific STS markers were detected and ‘−’ indicates that they were not.

### Causal genes for hybrid lethality exist in both the S and T subgenomes of *N. tabacum*

**Figure 2:**
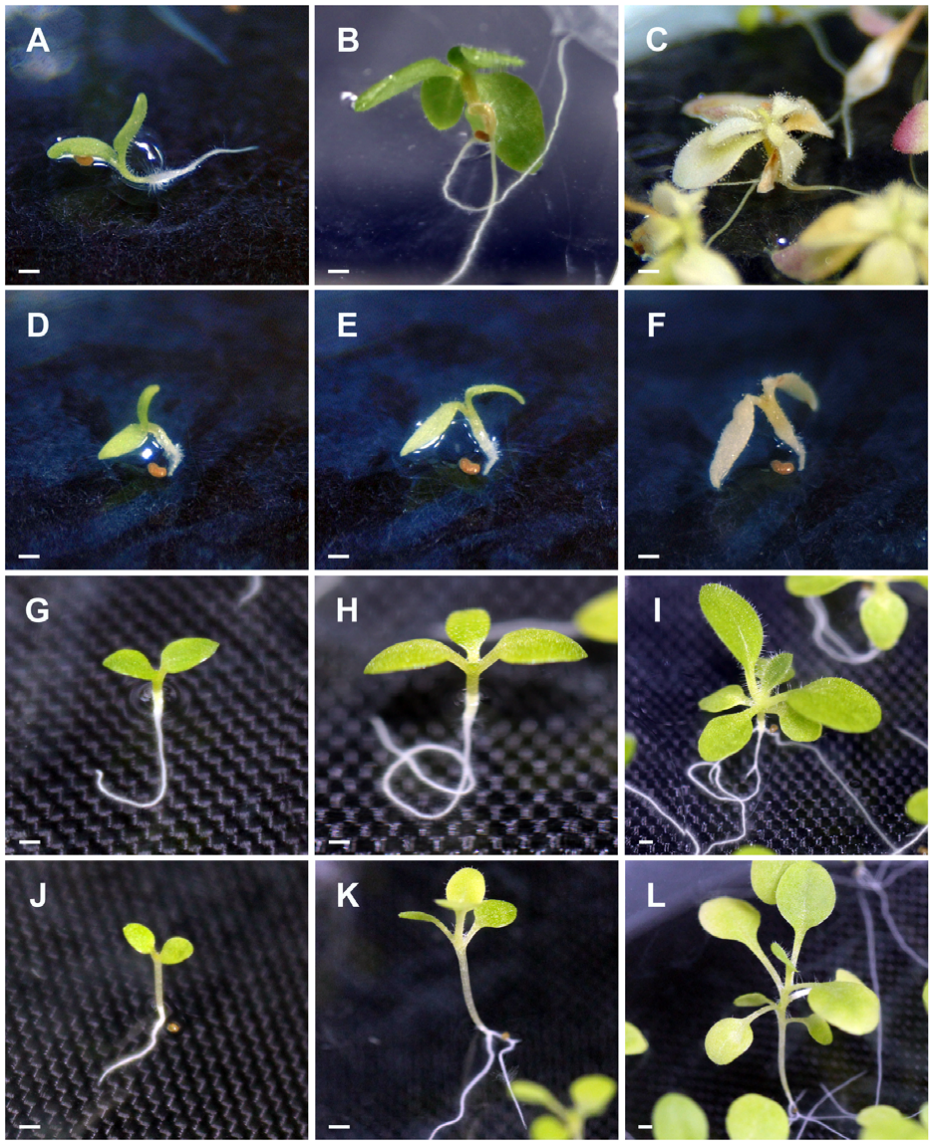
Lethal symptoms observed in hybrid seedlings from crosses between *N. occidentalis* and *N. tabacum* progenitors. (A–C) Hybrid seedlings from the cross *N. occidentalis* ♀×*N. sylvestris* ♂ at 24°C. The seedlings showed apparently normal growth at 3 days after germination (DAG) (A). The hypocotyl turned brown at 18 DAG (B), and the seedlings turned completely brown and died at 61 DAG (C). (D–F) Hybrid seedlings from the cross *N. occidentalis* ♀×*N. tomentosiformis* ♂ at 24°C. Although the seedlings exhibited apparently normal growth, root growth nearly stopped at 2 DAG (D), and the color of cotyledons and hypocotyl faded at 5 DAG (E). The seedlings turned completely brown and died at 16 DAG (F). (G–I) *N. sylvestris* grown at 24°C. *N. sylvestris* grew normally and did not show lethal symptoms at 3 (G), 10 (H), or 30 DAG (I). (J–L) *N. tomentosiformis* grown at 24°C. *N. tomentosiformis* also grew normally and did not display any lethal symptoms at 3 (J), 10 (K), or 30 DAG (L). Scale bars = 1 mm.

Two progenitors of *N. tabacum*, *N. sylvestris* and *N. tomentosiformis*, were crossed with *N. occidentalis* to reveal which subgenome of *N. tabacum* encodes gene(s) responsible for hybrid lethality. Conventional cross-pollination was successful when *N. occidentalis* was used as the maternal parent (Table 4). Seeds from two crosses, as well as those of *N. sylvestris* and *N. tomentosiformis*, germinated well at 28°C under *in-vitro* conditions. Hybrid seedlings and the parental species were grown at 24 or 28°C *in vitro*. At these two temperatures, all hybrid seedlings from both crosses died, whereas the parental species grew normally (Table 5, Figure 2). Lethal symptoms in hybrid seedlings of *N. occidentalis* ♀×*N. sylvestris* ♂ grown at 24°C appeared approximately 18 DAG as browning of the hypocotyl (Figure 2B). A few days later, chlorosis of leaves was observed. We identified this type of lethality as Type II. For hybrid seedlings of *N. occidentalis* ♀×*N. tomentosiformis* ♂, lethal symptoms at 24°C appeared as fading of shoot color at about 5 DAG and thus, the observed lethality was identified as Type V (Figure 2E). Within each cross, the same characteristic symptoms appeared at 24 and 28°C (Table 5).

**Table 4:** Conventional crossing between *N. occidentalis* and two progenitors of *N. tabacum*.

Cross combination	No. of flowers pollinated	No. of capsules obtained	No. of seeds sown	No. of hybrids obtained
*N. occidentalis* ♀×*N. sylvestris* ♂	20	19	160	119
*N. sylvestris* ♀×*N. occidentalis* ♂	20	0	–	–
*N. occidentalis* ♀×*N. tomentosiformis* ♂	17	10	157	140
*N. tomentosiformis* ♀×*N. occidentalis* ♂	20	0	–	–
*N. sylvestris*	–	–	120	117
*N. tomentosiformis*	–	–	120	114

**Table 5:** Viability of hybrid seedlings generated from crosses between *N. occidentalis* and two progenitors of *N. tabacum* at different temperatures.

		No. of hybrids			
Cross combination	Temperature (°C)	Total	Viable	Inviable	Lethality type^a^
*N. occidentalis* ♀×*N. sylvestris* ♂	24	58	0	58	II
	28	61	0	61	II
*N. occidentalis* ♀×*N. tomentosiformis* ♂	24	71	0	71	V
	28	69	0	69	V
*N. sylvestris*	24	59	59	0	–
	28	58	58	0	–
*N. tomentosiformis*	24	58	58	0	–
	28	56	56	0	–

a Type II, browning of hypocotyl and roots; Type V, fading of shoot color.

## Discussion

### Possible involvement of a dual lethal system causing hybrid lethality in crosses between *N. occidentalis* and *N. tabacum*

Hybrid seedlings of *N. occidentalis* ♀×*N. tabacum* ♂ and *N. occidentalis* ♀×*N. tomentosiformis* ♂ exhibited hybrid lethality that was characterized by the fading of shoot color. As this characteristic was distinct from those of the four known types of lethality in *Nicotiana* [2], we designated this type of hybrid lethality as Type V. Notably, Type V lethality is also temperature sensitive, similar to Types I, II, and III.

The type of lethality observed in the cross *N. occidentalis* ♀×*N. tabacum* ♂ (Type V) was different from that (Type II) typically observed in crosses between species of section *Suaveolentes* and *N. tabacum* [2], [22]. In addition to lethality type, the cause of hybrid lethality in *N. occidentalis* ♀×*N. tabacum* ♂ was also unique. As mentioned, we previously found that one or more genes on the Q chromosome in the S subgenome of *N. tabacum* were responsible for hybrid lethality in crosses of nine species in section *Suaveolentes* [20]–[22]. However, here, hybrid seedlings of *N. tabacum* ♀×*N. occidentalis* ♂ were inviable, even when they lacked the Q chromosome. Surprisingly, *N. occidentalis* yielded inviable hybrids after crossing with both progenitors of *N. tabacum*. Characteristic symptoms of hybrid lethality in the cross *N. occidentalis* ♀×*N. tabacum* ♂ were similar to those appearing in the cross *N. occidentalis* ♀×*N. tomentosiformis* ♂, strongly suggesting the involvement of gene(s) in the T subgenome. Nevertheless, we could not rule out the possibility that gene(s) in the S subgenome are also responsible for hybrid lethality in the cross *N. occidentalis* ♀×*N. tabacum* ♂. As lethal symptoms in the cross *N. occidentalis* ♀×*N. tomentosiformis* ♂ appeared faster than in the cross *N. occidentalis* ♀×*N. sylvestris* ♂, the phenotype of Type V lethality might be preferentially expressed in the cross *N. occidentalis* ♀×*N. tabacum* ♂. If this supposition is true, the hybrid lethality observed in the cross between *N. occidentalis* and *N. tabacum* is the first reported for a dual lethal system in the same cross-combination. Furthermore, the causal chromosome in the S subgenome of *N. tabacum* may be the Q chromosome, because hybrid lethality in the cross *N. occidentalis* ♀×*N. sylvestris* ♂ was Type II, which is often observed in crosses between species of section *Suaveolentes* and *N. tabacum* [2], [22]. Although further evidence is needed, we conclude that genes from both the S and T subgenomes of *N. tabacum* are responsible for hybrid lethality in the cross between *N. occidentalis* and *N. tabacum*.

The viability and reproductive fitness of hybrids are often compromised when they are derived from parents that differ in ploidy levels [37]. Such dosage-sensitive incompatibility in interploidy crosses arises in the absence of any allelic diversity and is under genetic control [37], [38]. Crosses between *N. occidentalis* and the two progenitors of *N. tabacum* were both interspecific and interploidal, because *N. occidentalis* is an allopolyploid, whereas *N. sylvestris* and *N. tomentosiformis* are diploids. Thus, it is difficult to determine whether hybrid lethality arose due to ploidy differences between the parental species. However, as we observed the identical hybrid lethality phenotypes in interploidy and same ploidy crosses, ploidy differences are not likely to have contributed to the observed hybrid lethality in crosses between *N. occidentalis* and the two progenitors of *N. tabacum*.

### Unilateral incompatibility in crosses between *N. tabacum* and *Suaveolentes* species

Unilateral incompatibility or unilateral incongruity (UI) is often observed in interspecific crosses in *Nicotiana*. In crosses between *N. tabacum* and Australian species of section *Suaveolentes*, including *N. occidentalis*, pollen of *Suaveolentes* species is often compatible with the *N. tabacum* pistil, but the reciprocal cross is incompatible. UI in *Nicotiana* arises from: (1) self-incompatibility [39], (2) differences in pistil length and pollen tube growth rate [40], [41], and (3) morphological abnormalities of pollen tubes, such as winding, swelling, and irregular callose deposition [42], [43]. Self-incompatibility is unlikely to be a factor in UI for crosses between *N. tabacum* and Australian species of section *Suaveolentes*, because both *N. tabacum* and *Suaveolentes* species are self-compatible. In crosses using *N. tabacum* as the female parent, pollen from six short-pistil species, *N. benthamiana*, *N. debneyi*, *N. goodspeedii*, *N. maritima*, *N. occidentalis*, and *N. velutina*, did not produce seeds, while pollen from three long-pistil species, *N. excelsior*, *N. gossei*, and *N. megalosiphon*, occasionally produced seeds (pollen from *N. tabacum* is compatible with pistils of all of these short- and long-pistil species) [21], [22], [33]. Therefore, pistil length and pollen tube growth rate might be involved in UI, although this is unlikely to be the only factor, as we observed that many flowers of long-pistil species dropped after pollination with *N. tabacum* pollen. Although morphological abnormalities of pollen tubes are observed in other crosses within the genus *Nicotiana*, we have no data on pollen tubes for the crosses between *N. tabacum* and *Suaveolentes* species.

### Evolutionary process of hybrid lethality in section *Suaveolentes*

Of the 12 *Suaveolentes* species used in the present and previous studies [20]–[22], [33], only three species (*N. occidentalis*, *N. benthamiana*, and *N. fragrans*) gave results different from those of the remaining 9 species. *N. benthamiana* and *N. fragrans* were the only species that produced viable hybrids after crosses with *N. tabacum*. This result is surprising, as section *Suaveolentes* presumably originated from a single polyploid event [26]. In a previous study, we concluded that the common gene(s) that trigger hybrid lethality by interaction with gene(s) on the Q chromosome should be widely distributed in species of section *Suaveolentes* [22]. Based on this conclusion, we suspect that the progenitor(s), namely *N. sylvestris* and/or section *Trigonophyllae*, which were involved in the formation of section *Suaveolentes*, might have possessed the gene(s) triggering hybrid lethality, whereas gene loss might have occurred during diploidization of the genome on polyploid establishment or during the subsequent divergence of *N. benthamiana* and *N. fragrans* [22]. However, it is also possible that the progenitors do not possess causal gene(s) for hybrid lethality in crosses with *N. tabacum*.

Information on phylogeny is critical to estimate the evolutionary order and timing of causal genetic changes underlying reproductive isolation [44]. In three phylogenetic trees respectively constructed by analyses of sequence data for ITS regions, plastid genes, and ncpGS, section *Suaveolentes* formed a monophyletic group with the African species, *N. africana*, as a sister to the remaining species of section *Suaveolentes* [14], [16], [27]. More recently, Marks et al. [45] reported the results of phylogenetic analyses of section *Suaveolentes* based on the combined data set of morphological characters, chromosome number, and nodes that received strong bootstrap support in trees constructed from individual analyses of sequences of ITS regions, plastid genes, and ncpGS. In their tree, which was constructed using *N. africana* as the functional outgroup, the South Pacific species *N. fragrans* was the basal lineage in the Australian and South Pacific clade of section *Suaveolentes*. In addition, the Australian species *N. debneyi* (New Caledonia, Lord Howe Island, and Eastern Australia) was a sister species to all other endemic Australian species, including *N. occidentalis* and *N. benthamiana*.

**Figure 3:**
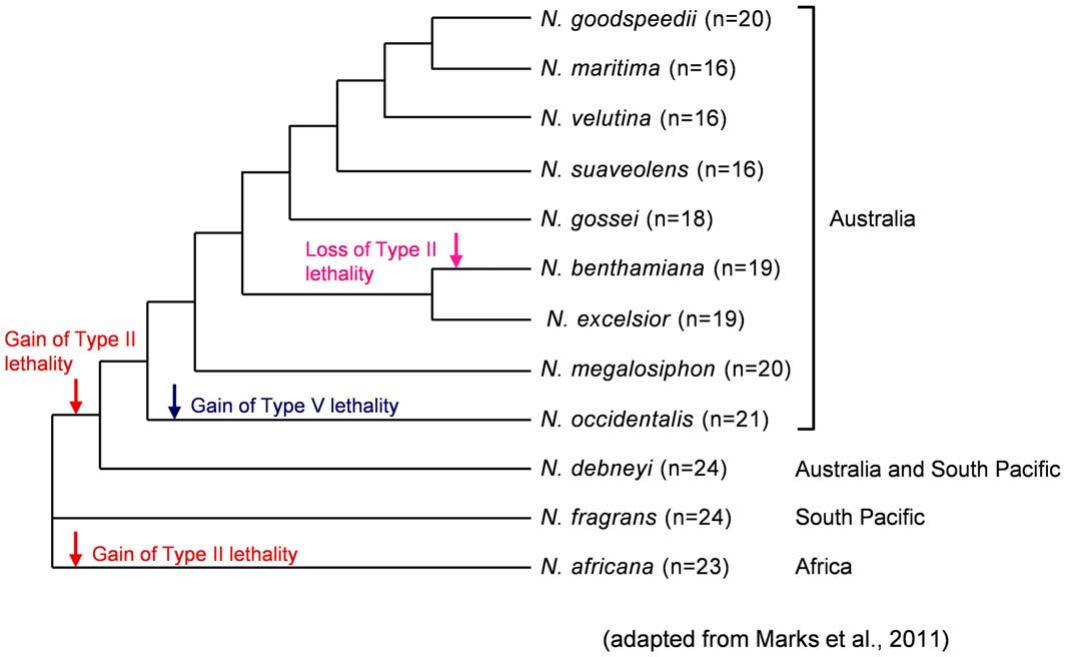
Schematic phylogenetic tree showing gain and loss of hybrid lethality in section *Suaveolentes*. The schematic phylogenetic tree including 12 *Suaveolentes* species used in our studies on hybrid lethality was drawn based on tree by Marks et al. [45]. Gains of Type II lethality were established in the lineage leading to *N. africana* and the lineage leading to Australian clade. Subsequently, gain of Type V lethality was established in the lineage leading to *N. occidentalis*. In the lineage leading to *N. benthamiana*, loss of Type II lethality was established.

Taking into account the results of recent phylogenetic analyses [45] and the crosses with *N. tabacum* performed here, we propose a new model for the evolutionary process leading to hybrid lethality in section *Suaveolentes* (Figure 3). It has been reported that the allotetraploid ancestor of the section, of which the maternal and paternal progenitors are *N. sylvestris* and section *Trigonophyllae*, respectively, occurred in South America [16], [27]. In our proposed model, we considered that the allotetraploid ancestor did not cause hybrid lethality after crosses with *N. tabacum*. The appearance of Type II lethality in the lineages leading to *N. africana* and the Australian clade possibly occurred after the divergence of *N. africana*, *N. fragrans*, and the remaining species in section *Suaveolentes* (Figure 3). However, we cannot deny the possibility that Type II lethality appeared in the allotetraploid descendent after the divergence of *N. fragrans*, and the allotetraploid descendent subsequently dispersed to Africa and Australia. Type II lethality may be caused by interaction with the *Hla1-1* or other alleles found in the *HLA1* locus, which was originally identified in *N. debneyi* [33], and gene(s) on the Q chromosome of *N. tabacum*. In Australia, an explosive radiation of taxa occurred that was largely accompanied by dysploid reductions, likely due to chromosomal fusions [27]. During the process, additional genetic changes that reinforced postzygotic isolation with *N. tabacum* accumulated in the lineage leading to *N. occidentalis*, leading to the appearance of Type V lethality (Figure 3). Conversely, loss of Type II lethality gave rise to the lineage leading to *N. benthamiana*.

Although the considerable amount of accumulated phylogenetic data for section *Suaveolentes* has allowed us to propose an evolutionary model of hybrid lethality, the present data are insufficient to definitively determine the origin and interspecies relationships of this section. The accumulation of additional phylogenetic data and a greater understanding of the mechanisms underlying hybrid lethality will allow further clarification of how hybrid lethality has evolved through speciation in section *Suaveolentes*.

## Materials and Methods

### Plant materials

*N. tabacum* (2n = 48, SSTT) ‘Red Russian’ and ‘Samsun NN’, *N. sylvestris* (2n = 24, SS), and *N. tomentosiformis* (2n = 24, TT) were used as parents for reciprocal crosses with *N. occidentalis* (2n = 42). *N. tabacum* monosomic plants (2n = 47) were also used for crossing with *N. occidentalis*. The monosomic plants were identified from F_1_ plants from the cross of *N. tabacum* Haplo-Q ♀×‘Samsun NN’ ♂ using Q-chromosome-specific STS marker QCS1 [35]. All plants were cultivated in a greenhouse.

### Interspecific crosses

Conventional crossing and sowing were carried out as follows: flowers of plants used as maternal parents were emasculated one day before anthesis and pollinated with the pollen of paternal parent plants. F_1_ seeds were soaked in a 0.5% gibberellic acid (GA_3_) solution for 30 min and sterilized with 5% sodium hypochlorite for 15 min. The sterilized seeds were sown in Petri dishes (60-mm diameter, 17-mm depth) containing 8 ml of 1/2 MS medium [46] supplemented with 1% sucrose and 0.2% Gelrite (pH 5.8), and then cultured at 28°C under continuous illumination (approximately 150 µmol m^−2^ s^−1^).

Test-tube pollination in combination with ovule culture was performed as previously described [47] to obtain hybrid seedlings between *N. tabacum* monosomic plants and *N. occidentalis*. Anthers of *N. occidentalis* plants used as paternal parents were aseptically excised from still-closed flowers and stimulated to dehisce in an incubator held at 28°C. Flowers of monosomic plants used as maternal parents were emasculated one day before anthesis. On the next day, flowers of monosomic plants were collected and their corolla, sepals, and styles were removed. The ovaries were surface-sterilized with 70% ethanol for 30 s followed by a 5% sodium hypochlorite solution for 5 min. The ovary walls were peeled back to expose the placentas with intact ovules and the ovaries were then placed in Petri dishes containing 8 ml of medium supplemented with 3% sucrose and 0.8% agar (pH 5.8). Pollen of *N. occidentalis* was spread on the surface of the placentas, which were then maintained at 28°C under continuous illumination. Fertilized and enlarged ovules were excised from placentas 10 to 14 days after pollination and cultured in Petri dishes containing 8 ml of 1/2 MS medium supplemented with 3% sucrose and 0.8% agar (pH 5.8) at 28°C under continuous illumination.

### Cultivation of hybrid seedlings

Hybrid seedlings and parental species were cultured at 24 or 28°C under continuous illumination. Several seedlings were transferred to flat-bottomed test tubes (25-mm diameter, 100-mm length) that contained 10 ml of 1/2 MS medium supplemented with 1% sucrose and 0.2% Gelrite (pH 5.8) immediately after germination and were cultured at 24, 28, or 34°C under continuous illumination. Seedlings cultured at 34°C for 30–60 DAG were then further cultured at 28°C under continuous illumination.

### PCR analysis

Total DNA was extracted from the leaves of each plant using a cetyltrimethylammonium bromide-based method [48]. Four Q-chromosome-specific STS markers, QCS1, QCS2, QCS3, and QCS4 [20], [35], were detected by PCR as follows. Reaction mixtures consisted of 1× ThermoPol reaction buffer (New England Biolabs, Tokyo, Japan), 0.2 mM each dNTP, 0.2 µM each primer, 20 ng template DNA, and 0.5 U *Taq* DNA polymerase (New England Biolabs) in a total volume of 20 µl. PCR amplification was performed using a PC-818A Program Temp Control System (Astec, Fukuoka, Japan) programmed for 3 min at 94°C for initial denaturation, followed by 35 cycles of 30 s at 94°C, 30 s at 60°C, and 30–90 s at 72°C, with a final 5-min extension at 72°C. PCR products were separated by electrophoresis in 1.5% agarose gels with TBE buffer and were then visualized by staining with ethidium bromide.

### Ethics

The ethics committee of Osaka Prefecture University specifically waived the need for consent.
